# Time for an Australian and New Zealand randomized controlled trial to study the modified Kono S anastomosis

**DOI:** 10.1111/ans.17991

**Published:** 2022-12-05

**Authors:** David A. Clark, Nicholas Smith, Imogen Clark, Hugh Giddings, Ruben Rajan

**Affiliations:** ^1^ Department of Surgery Royal Brisbane and Women's Hospital Brisbane Queensland Australia; ^2^ Faculty of Medicine University of Qld, Hawkins Drive Queensland Brisbane Australia; ^3^ Department of Surgery St Vincent's Private Hospital Northside Brisbane Queensland Australia; ^4^ Department of Surgery Waikato Hospital Hamilton New Zealand; ^5^ Faculty of Medicine and Health University of Sydney Sydney New South Wales Australia; ^6^ Faculty of Medicine and Health Surgical Outcomes Research Centre (SOuRCe) Camperdown New South Wales Australia; ^7^ Department of Surgery Royal Perth Hospital Perth Western Australia Australia

There is growing and compelling evidence supporting the Kono S anastomosis, after an ileocolic resection for Crohn's disease, to see a reduction in recurrent ileal Crohn's disease.^1^ The principles of this operation include a close intestinal mobilization that preserves the mesentery and neurovascular supply, and a 7 cm anti‐mesenteric transverse ‘stricturoplasty‐like’ anastomotic configuration. The stapled ends are secured together to create a supporting column.^2^

Crohn's disease is a chronic inflammatory bowel disease that occurs in a genetically susceptible individual in response to an unknown environmental stimulus. The incidence in Western populations is increasing and the prevalence in Australia has been estimated at 306 per 100 000.^3^ It is estimated that 80% of patients with Crohn's disease require surgery at some point in their life.^2^ After surgery, endoscopic recurrence (ER) is common with up to 90% of patients found to have ER at 12 months.^4^, ^5^ Symptomatic recurrence is observed at 28% and 36% at 5 and 10 years postoperative.^6^ Contemporary data have shown a reduction in the need for further surgery, but still patients required a second operation at 17.7% and 31.3% at 5 and 10 years respectively.^7^

Numerous studies have investigated the handsewn end to end anastomosis (ETEA) against the stapled side‐to‐side anastomosis (STSA). The stapled STSA has been shown to have lower anastomotic complications and either a lower recurrence rate or no difference when compared with the handsewn ETEA.^8^, ^9^ The ETEA may facilitate subsequent colonoscopic evaluation. The preference for anastomotic configuration amongst Australian and New Zealand (ANZ) colorectal surgeons is unknown.

In 2003, Toru Kono and his colleagues introduced the Kono S anastomosis. In the 2015 paper the Japanese group, along with one American centre reported the safety of the technique in 187 patients and extremely low surgical recurrence rates of 1.4% at 5 years follow‐up.^10^ Shimsada *et al*. reported a comparative study of the Kono S and ETEA in 2018, analysing 215 patients. The patients were not randomized but those operated on after 2009 underwent a Kono S anastomosis. Prior to 2009, the patients underwent a handsewn ETEA. The 5 year surgery‐free survival rate was significantly higher in the Kono S group versus the ETEA group (95% vs. 81.3%; *P* < 0.001).^11^

Further robust evidence of the value of the Kono S anastomosis was presented in 2021 when Luglio *et al*. reported the findings of the SuPREMe‐CD Study. This was a randomized controlled trial (RCT) of 79 patients comparing the Kono S with the conventional stapled STSA and was powered to show a difference assuming a >30% reduction in endoscopic recurrence. At the six‐month endoscopic assessment, recurrence (Rutgeerts >i2) was seen in 22.2% in the Kono group and 62.8% in the STSA group (*P* < 0.001, odds ratio 5.91). There was a commensurate longer time to clinical recurrence in the Kono S group (hazard ratio 0.36, *P* = 0.037).^1^

At the same time as the Luglio *et al*. study of Kono S, and its mesenteric preservation, Coffey *et al*. investigated the effect of the extent of mesenteric resection on recurrence rates in Crohn's disease. The authors of Coffey *et al*. found that inclusion of the mesentery in the resection led to lower surgical recurrence compared with mesenteric conservation in their cohort of 64 patients (2.9 vs. 40%; *P* = 0.003), followed up for a mean of between 51.7 and 69.9 months.^12^

Given these opposed approaches to the mesentery in the literature, and to ascertain the preferences for anastomotic configuration and extent of mesenteric resection after ileocolic surgery for Crohn's disease amongst ANZ surgeons, a society‐approved survey was distributed. One hundred and thirty‐four responses were received (39.7%). The preferred anastomotic configuration was the stapled STSA (68, 50.7%), followed by the ETEA (27, 20.1%) (Fig. 1a). Thus, the stapled STSA could constitute the preferred control group in an RCT. Twenty‐one (15.7%) of respondents preferred the Kono S anastomosis. Of these, only four respondents were using the close intestinal dissection plane that preserves the mesentery, as described in the technical papers. The majority, of those performing the Kono S, (14 of 21, 66.7%) resected at the proximal extent of the abnormal mesentery. Overall, 101 (75.4%) of respondents would transect the mesentery at the proximal junction of the abnormal and normal mesentery (Fig. 1b). Thus, this information informs the intervention group preferably to be a modification of the Kono S technique.

**Fig. 1:**
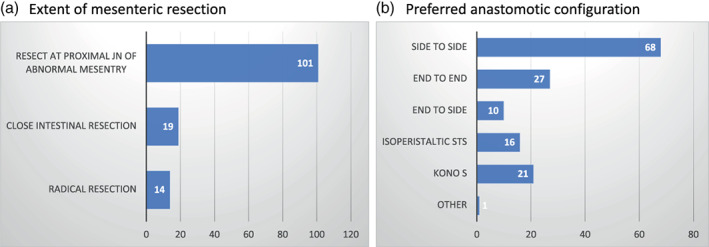
(a) Extent of mesenteric resection. (b) Preferred anastomotic configuration. Count on x‐axis; JN, junction; STS, side to side.

Holubar *et al*. modified the Kono S anastomosis by including the mesentery and called this approach, Mesenteric Excision and Exclusion (MEE); combining Kono S and mesenteric resection.^13^ In this Cleveland Clinic study of 22 patients, the authors reported that the approach was safe and highly feasible.

An ANZ RCT comparing the modified Kono S anastomosis (involving resection of the mesentery at the junction of normal and abnormal portions) with the stapled STSA, observing the same approach to the mesentery, would answer the question of which anastomotic configuration sees to the lowest incidence of recurrent Crohn's disease. This present study would indicate that there should be equipoise among ANZ colorectal surgeons. The proposed RCT is registered on the ANZ Clinical Trials Registry (ACTRN12622000809730) and all interested centres are invited to participate.

## Author contributions

**Nicholas Smith:** Conceptualization; writing – review and editing. **Imogen Clark:** Writing – original draft; writing – review and editing. **Hugh Giddings:** Writing – original draft; writing – review and editing. **Ruben Rajan:** Conceptualization; writing – original draft; writing – review and editing. **David A. Clark:** Conceptualization; data curation; formal analysis; funding acquisition; investigation; methodology; project administration; resources; supervision; validation; visualization; writing – original draft; writing – review and editing.

## Ethical approval

Human research and ethics approval was granted by the St Vincent's Health and Aged Care Human Research and Ethics Committee [EC00324]; HREC_22‐01_DCLA.
